# High Prevalence of *atpE* Mutations in Bedaquiline-Resistant *Mycobacterium tuberculosis* Isolates, Russia

**DOI:** 10.3201/eid3103.241488

**Published:** 2025-03

**Authors:** Danila Zimenkov, Anastasia Ushtanit, Elizaveta Gordeeva, Elena Guselnikova, Yakov Schwartz, Natalia Stavitskaya

**Affiliations:** Center for Precision Genome Editing and Genetic Technologies for Biomedicine, Engelhardt Institute of Molecular Biology, Russian Academy of Sciences, Moscow, Russia (D. Zimenkov, A. Ushtanit); Federal State Budgetary Institution Novosibirsk TB Research Institute, Ministry of Health, Novosibirsk, Russia (E. Gordeeva, E. Guselnikova, Y. Schwartz, N. Stavitskaya)

**Keywords:** tuberculosis and other mycobacteria, bacteria, antimicrobial resistance, bedaquiline, determinants of resistance, drug-resistant tuberculosis, *atpE*, ATP synthase, mmpL3-mmpL4 efflux, Russia

## Abstract

Bedaquiline is a cornerstone drug for treating drug-resistant tuberculosis. We analyzed 11 isolates from 9 patients who were treated with a bedaquiline-based regimen and remained culture-positive long after treatment start. In 4 of 8 resistant isolates, we found substitutions in AtpE, which encodes subunit *c* of the *Mycobacterium tuberculosis* ATP synthase and is rarely identified in clinical isolates. We found Ile66Met and Glu61Asp substitutions in 2 cases each. Additional mutations in *mmpL5*, *mmpL4*, and *atpB* genes could affect the susceptibility to bedaquiline. MmpL5(Asn772Thr) emerged during bedaquiline treatment, whereas AtpB(Val165Leu) was found in 1 case simultaneously with the loss-of-function *mmpR5* mutation in a susceptible strain. The loss-of-function mutation in the *mmpL4* efflux gene was identified in the mixed state, pointing to ongoing selection in a bedaquiline-resistant isolate. Another case of the emergence of the *mmpL4* mutation, accompanied by a proportional increase in bedaquiline MIC, was identified by retrospective analysis of genomes from bedaquiline-resistant isolates.

Drug-resistant tuberculosis (TB) remains a major problem of the public health system. In 2022, TB was newly diagnosed and officially notified in ≈7.5 million persons, and the total number of deaths caused by TB was 1.30 million. In 2019, TB was the 13th leading cause of death worldwide and the leading cause of death from a single infectious agent (*1*). The incidence of drug-resistant TB is estimated to be >400,000 new cases (*1*).

The limited number of effective TB drugs has pushed the development of new candidates. Bedaquiline, introduced in 2012, began the new era of therapy and was successfully used in combinations with linezolid, clofazimine, and pretomanid/delamanid in new regimens, including all-oral short-course regimens for drug-resistant TB (*2*).

Bedaquiline has a novel distinct mode of action on bacterial physiology by blocking the ATP synthase of *Mycobacterium tuberculosis*, thus causing the decrease of the ATP pool (*3*) and consequent cell death (*4*). Two main mechanisms of bedaquiline resistance by alteration of the target and drug efflux were identified in vitro (*3*,*5*) and, subsequently, in clinical isolates (*6*–*9*).

In the first main mechanism of resistance, the iron-scavenger siderophore transporter complex MmpS5/L5 (*10*) provides bedaquiline efflux from the cytoplasm. Mutations in the repressor gene *mmpR5*(*rv0678*) lead to derepression of the operon *mmpS5-mmpL5*, thus lowering the bedaquiline concentration inside the cell (*5*). Mutations are spread along the open reading frame and include insertions, deletions, premature stop-codons, and amino acid substitutions (*11*). The most prevalent type of mutations is nucleotide insertion or deletion in homopolymeric sequences in the gene (*12*). Drug-resistant isolates with *mmpR5* mutations that emerged during bedaquiline treatment have been described in many regions of the world (*13*–*15*).

The second type of mutations leading to bedaquiline resistance occur in the *c* subunit of ATP synthase, encoded by the *atpE* gene (*3*). Amino acid substitutions at particular positions in the protein prevent the drug binding (*16*) and are rarely identified in clinical strains (*12*). Isolates with *atpE* mutations possess higher bedaquiline MICs and are supposed to emerge after repressor-inactivating mutations in clinical strains (*17*).

Other genetic traits have been proposed to cause bedaquiline resistance; however, the number of such cases is low, and the World Health Organization (WHO) has associated substitutions in only *atpE*, *mmpR5*, and *pepQ* genes with resistance to bedaquiline (*18*). Most of those mutations are in interim status because of insufficient statistics of characterized isolates with a particular mutation. Only the combined category of loss-of-function mutations in the *mmpR5* gene and selected frameshifting mutations in hot spots are classified as category 1 mutations. WHO experts indicate that the functionality of the *mmpS5* and *mmpL5* genes should also be validated because of epistatic interactions with repressor mutations (*19*). In this study, we analyzed bedaquiline-resistant clinical strains isolated at Novosibirsk TB Research Institute (Novosibirsk, Russia), analyzed mutation profiles using whole-genome sequencing, and proposed the participation of novel determinants of bedaquiline resistance. The study protocol was approved by the Ethical Committee of the Federal State Budgetary Institution Novosibirsk TB Research Institute (protocol no. #52/1, May 5, 2021).

## Methods

### Clinical Isolates and Drug Susceptibility Testing

Clinical isolates were obtained from patients of the Novosibirsk TB Research Institute in whom TB had first been diagnosed during 2006–2018 (Table 1). In the study period (2021–2022), those patients received treatment with a bedaquiline and linezolid–based regimen. Isolates were obtained using liquid media in the Bactec MGIT 960 system (BD, https://www.bd.com) and were further used for drug susceptibility tests, storage, and DNA isolation for molecular tests.

**Table 1:** Clinical characteristics of TB cases and resistance to bedaquiline and linezolid determined by phenotypic and genotypic methods in study of high prevalence of *atpE* mutations in bedaquiline-resistant *Mycobacterium tuberculosis* isolates, Russia*

Category	Isolate identification								
	#1	#2	#3	#4	#5	#6	#7	#8	#9c
Year of TB diagnosis	2017	2006	2015	2010	2018	2017	2017	2012	2007
HIV status	Yes	No	No	No	Yes	No	No	Yes	No
Source of the culture	Sputum	Surgery	Sputum	Wound exudate	Sputum	Sputum	Wound exudate	Sputum	Sputum
No. days from treatment start	92	718	196	1,045	206	558	866	609	1,154
Treatment outcome	Treatment failed	Not evaluated	Treatment failed	Death from TB	Death from TB	Treatment failed	Treatment failed	Treatment failed	Treatment failed
Sublineage	B0	B0	B0	CA/R	B0	B0	B0	B0	B0
Bedaquiline phenotype	R	R	R	S	R	R	R	R	R
AtpE Glu61Asp, g>t							100%		
AtpE Glu61Asp, g>c									59%
AtpE Ile66Met					100%			100%	
MmpR5 Cys46fs						21%			
MmpR5 Asp47fs	52%								
MmpR5 Glu49fs	42%			97%					
MmpR5 Ser63Gly			75%						
MmpR5 Ile67fs		97%							96%
MmpR5 Gln76stop						28%			
MmpR5 Leu143_ Glu147dup						22%			
MmpL4 Leu130fs†		91%							
MmpL5 Asn772Thr†									37%
AtpB Val165Leu†				100%					
Linezolid phenotype	S	S	S	R	S	S	S	R	R
RplC Cys154Arg				100%				100%	100%

*Percentages indicate relative number of reads with mutations. ID, identification; R, resistant; S, susceptible; TB, tuberculosis. †Mutations in candidate genes associated with resistance to bedaquiline.

We performed bedaquiline and linezolid susceptibility tests using the modified proportional method in the automatic Bactec MGIT 960 system. Russia’s national guidelines for TB treatment are in accordance with WHO guidelines (*20*), and we used currently approved critical concentrations for bedaquiline and linezolid of 1 mg/L for both tests. We dissolved and diluted bedaquiline (Molekula Ltd., https://molekula.com) in DMSO and added 100 μL per MGIT tube. We dissolved linezolid (Glenmark Pharmaceuticals, https://glenmarkpharma.com) in sterile H_2_O as recommended in the guidelines.

### Whole-Genome Sequencing

The strains for whole-genome sequencing were recultured on solid Lowenstein-Jensen medium for ≈4 weeks at 37°C and then heat inactivated. We extracted genomic DNA using the Gentra Puregene Yeast/Bact. Kit B (QIAGEN, https://www.qiagen.com). We prepared the DNA libraries using the Illumina DNA Prep kit and performed sequencing using the MiniSeq High Output Kit (300 Cycles) on the MiniSeq platform (Illumina, https://www.illumina.com).

We analyzed sequencing data in FastQ format using the Galaxy web platform (https://usegalaxy.org). We trimmed the reads using the Trimmomatic tool (https://toolshed.g2.bx.psu.edu/repository?repository_id=ef9e620e9ac844b3), mapped them to the reference genome of *M. tuberculosis* H37Rv (GenBank accession no. NC_000962.3) with BWA-MEM2 (https://toolshed.g2.bx.psu.edu/repository?repository_id=48f9d7927f0fd013) , and refined using BamLeftAlign (https://toolshed.g2.bx.psu.edu/repository?repository_id=903c3759b76db034). We performed variant calling using FreeBayes (https://toolshed.g2.bx.psu.edu/repository?repository_id=491b7a3fddf9366f) and filtered using the VCFlib toolkit (https://toolshed.g2.bx.psu.edu/repository?repository_id=db548aefa5e7e768). The variants annotation was performed using SnpEff (https://toolshed.g2.bx.psu.edu/repository?repository_id=93a5efea7e957b53). We performed further bioinformatic analysis of the obtained single-nucleotide polymorphisms with custom Python scripts. Alternatively, we used the genome of *M. tuberculosis* B0/W148 (GenBank accession no. CP012090.1) as a reference for the second round of alignment of raw reads for the validation of mutations located in highly repetitive genomic loci.

### Analysis of the CRyPTIC Database

For phylogenetic and mutation frequency analysis, we used a dataset consisting of 9,941 of 12,288 isolates that were sequenced and analyzed by the CRYpTIC Consortium (*21*). The remaining 2,347 isolates had an ambiguous description of amino acid substitutions and were omitted. We built the phylogenetic tree by nearest-neighbor method in MEGA 11 software (https://www.megasoftware.net) using the pairwise distances calculated from genome mutations. We omitted highly repetitive PE, PPE, PE-PGRS genes, and insertion elements (Appendix Figure). We verified the reliability of the phylogenetic tree by clonal distribution of selected lineage-specific single-nucleotide polymorphisms. We assembled data consisting of the phylogenetic tree, isolate susceptibility profiles, and mutation profiles in a local database, powered by custom Python scripts.

## Results

### Molecular Determinants of Resistance to Bedaquiline and Linezolid

During June 2021–May 2022, we identified 9 cases of culture positivity long after the initiation of treatment with bedaquiline plus linezolid–based regimens. We performed drug susceptibility tests for bedaquiline and linezolid using the proportion method. Of the 9 isolates, 8 were resistant to bedaquiline; 2 of the 8 bedaquiline-resistant isolates and the 1 bedaquiline-susceptible isolate were resistant to linezolid (Table 1).

In all 9 isolates, including the susceptible isolate, we identified mutations in either *mmpR5* or *atpE* genes (Table 1). Most of the mutations identified were in mixed state with wild-type sequence, so the allele frequencies were estimated by calculating the relative number of reads with mutations. Unexpectedly, 4 of the 8 resistant strains had substitutions in 61 and 66 codons of the *atpE* gene, which are rarely reported in bedaquiline-resistant isolates. The 100% mutated allele frequency at the *atpE* locus correlated with the absence of substitutions in the *mmpR5* gene in 3 isolates (#5, #7, and #8). Another isolate (#9c) in addition to AtpE Glu61Asp in the mixed state also had the loss-of-function frameshifting MmpR5 Ile67fs and substitution MmpL5 Asn772Thr, which could affect bedaquiline resistance.

In 6 of 9 isolates, we observed variable mutations of *mmpR5*, predominately leading to frameshifts. Single-nucleotide insertions were located in 2 hot spots: around codons 46–49 (3 cases) and 67 (2 cases). Another type of mutations, the amino acid substitution Ser63Gly in MmpR5, was observed only in 1 case with an allele frequency of ≈75%. Serine 63 is located in the turn part of the HTH DNA binding domain of the MmpR5 repressor (*22*), providing the rationale for the effect of substitutions at this point on bedaquiline resistance. One isolate susceptible to bedaquiline had an inactivating mutation in the *mmpR5* gene with an allele frequency close to 100% and mutation Val165Leu in the *atpB* gene encoding the *a* subunit of ATPsynthase and located just upstream of the *atpE* gene (Table 1). This isolate belongs to the Central Asia/Russian genotype, contrary to Beijing B0/W148 for all other isolates. Resistance to linezolid in 3 isolates was caused by the canonical substitution Cys154Arg in the ribosomal protein RplC (*23*).

### Candidate Markers of Resistance to Bedaquiline

We also sequenced 3 consequent isolates from the same patient, who was treated with a bedaquiline-based regimen. Although they were isolated during only half a year, we observed the dynamic change in *mmpR5* mutations and the emergence of AtpE(Glu61Asp) amino acid substitution (Table 2). Thus, in the latest sample, allele frequency of AtpE(Glu61Asp) was 59%, and MmpR5(Ile67fs) was ≈100%. Another mutation MmpR5(Leu143stop) was highly represented in the first sample in the series and then gradually disappeared. Of note, we observed that the additional substitution in the efflux pump subunit MmpL5(Asn772Thr) emerged simultaneously with the AtpE substitution.

**Table 2:** Allele frequencies change in sequential isolates from patient #9 during treatment in study of high prevalence of *atpE* mutations in bedaquiline-resistant *Mycobacterium tuberculosis* isolates, Russia

Gene or intergenic region	Amino acid substitution	Isolate 9a, day 1,004	Isolate 9b, day 1,037	Isolate 9c, day 1,154	Associated with resistance
*atpE*	Glu61Asp			59%	Bedaquiline
*mmpR5 (Rv0678)*	Ile67fs	18%	15%	96%	Bedaquiline
	Leu143stop	80%	63%		
*mmpL5*	Asn772Thr			37%	Bedaquiline
*alr*	Glu6Gln	78%	65%		D-cycloserine
*gabD2*	Val153fs			39%	D-cycloserine
*Rv2690c*	Gly191Arg			26%	(Pyrazinamide)
*pncA*	Met1Thr	100%	100%	100%	Pyrazinamide
*ceoC*	Ala130Thr			25%	(Isoniazid)
*PPE8*	Asp1235fs	64%	63%		
*PPE35*	Leu939fs		18%	96%	
*Rv2298*	Gly16Glu			23%	
*moeW-mmpL9*		71%	64%		
*sigL*	Leu120Leu			63%	
*purM*	Pro290Pro			22%	
*Rv3083*	Leu439Leu		6%	22%	

Allele frequencies of mutations associated with resistance to other drugs and mutations in genes possibly associated with virulence and host-adaptation also changed in sequential isolates. Furthermore, 3 synonymous amino acid substitutions were recorded in the latest isolate #9c.

In addition to the isolates from case #9, isolates #2 and #4 also had mutations that could be associated with bedaquiline resistance. In those cases, the reference susceptible strains isolated before the start of the treatment were not available, and the emergence of mutations cannot be confirmed directly. The bedaquiline susceptible isolate #4 had Val165Leu substitution in *a* subunit of ATPase, encoded by the *atpB* gene, in addition to the loss-of-function MmpR5(Glu49fs) (Table 1). Another bedaquiline-resistant isolate (#2) had a frameshifting mutation in the *mmpL4* gene together with a loss-of-function mutation in *mmpR5*.

## Discussion

Resistance of *M. tuberculosis* to bedaquiline is driven by 2 main mechanisms, drug efflux by the MmpS5-MmpL5 complex and alteration of its binding site in the rotor part of ATP synthase (*3*,*5*). Mutations in both repressor gene *mmpR5* and *atpE* encoding the *c* subunit emerge during bedaquiline treatment. Mutations in *mmpR5* are distributed along the open reading frame, lead to an increase in MIC, and are found more frequently in clinical strains.

We identified a high prevalence of the *atpE* mutations in clinical isolates from patients who were treated with the bedaquiline-linezolid treatment scheme and remained culture-positive long after the start of the treatment. Most isolates developed phenotypic resistance to bedaquiline detected by Bactec MGIT 960 with a recommended critical concentration of 1 mg/L. Four of 8 bedaquiline-resistant strains had amino acid substitutions Glu61Asp or Ile66Met of the *c* subunit in positions that are responsible for the direct interaction with bedaquiline. Those substitutions are classified by the WHO as associated with resistance-interim (*18*).

Only 17 clinical MTB isolates with *atpE* mutations have been described in 9 studies (*9*,*12*,*24*–*30*). Another study from China also describes the existence of AtpE Asp28Gly and Ala63Pro substitutions in clinical strains resistant to bedaquiline; however, the number of such isolates cannot be extracted from the published data (*31*). The most frequent mutation in AtpE was Ile66Met, found in 6 cases. The same dominance was also found in this study: 2 of 4 isolates bore the same substitution. It could be assumed that this mutation provides the best balance between loss of susceptibility to bedaquiline and fitness cost of altered structure of ATP synthase. However, in a report from France, such *atpE* mutation was transient in a series of clinical isolates from 1 patient and was lost in subsequent isolates; the patient was successfully cured (*28*).

The effect of particular mutations in *mmpR* and *atpE* on the bedaquiline MIC value is variable. Part of mutations in the *mmpR5* repressor gene lead to a modest increase in MIC close to the borderline value and thus are considered not associated with resistance (*32*). Of note, even loss-of-function mutations in the repressor, which are supposed to lead to complete derepression of the efflux operon *mmpS5-mmpL5*, do not invariantly lead to resistance, as was found for an isolate in this study. In this case, the determination of MIC could have been particularly beneficial to estimate the effect of mutation on phenotype; the proportion method with currently approved critical concentration definitely limits the study.

Other genetic traits could affect the phenotype by epistatic interaction, as was shown for the isolate with both derepression of the efflux operon and the inactivated efflux gene (*19*). In general, the particular genotype of the pathogen could have an effect not only on transmission dynamics but also on the speed of resistance acquisition and survival under drug-induced stresses (*33*). In Russia, 2 main genotypes of lineage 2 are associated with resistance to many drugs and rapid transmission in the population, B0/W148 and Central Asia/Russia (*34*). At least 12 of 17 previously reported *atpE* mutants belong to either of these sublineages; 10 of them were reported in different regions of Russia. Only 2 strains belong to lineage 4 (*24*) and lineage 3 (*27*). All bedaquiline-resistant isolates reported here belong to Beijing B0/W148, whereas the single susceptible isolate belongs to the Beijing Central Asia/Russia sublineage.

The recommended critical concentration of 1 mg/L for resistance determination using the Bactec MGIT 960 is probably too high (*32*). A substantial overlap of MIC values at 0.125 mg/L and 0.25 mg/L was previously reported for pairs of exposed and nonexposed and wild-type and mutated isolates (*12*). On the basis of those observations, the critical concentration should be lowered to at least 0.25 mg/L for better differentiation of resistant and susceptible isolates. The current value enables identification of strains with substantially elevated MIC, whereas intermediate resistance is missed by the phenotypic method. Thus, the detection of a considerable number of isolates with AtpE substitutions, which are associated with high bedaquiline MICs, was not unexpected. In addition, we identified strains with *mmpR5* mutations only, including both frameshifting and amino acid substitutions such as Ser63Gly, which occurred in the HTH DNA binding domain of the repressor.

Contrary to several previous reports, we did not find *pepQ* or *rv1979c* mutations in bedaquiline-resistant isolates (*15*,*35*–*37*). Although the isolated mutations in *pepQ* are rare and usually are accompanied with *mmpR5* mutations, WHO has stated that *pepQ* mutations are associated with resistance (*18*). We propose that *mmpL5*, *mmpS4-mmpL4*, and *atpB* genes could also affect the bedaquiline-resistance phenotype from the analysis of microevolution events and the identification of mutations in single isolates in heteroresistant state, which also point to the ongoing evolution of the pathogen.

We observed mutations in genes that encode the siderophore export transport proteins MmpL5/S5 and MmpL4/S4 (*10*). First, the amino acid substitution Asn772Thr of the efflux MmpL5 emerged in the latest of series of 3 consequent isolates from 1 patient, together with fixation of loss-of-function variant of the *mmpR5* gene and the emergence of AtpE Glu61Asp. The *mmpR5* mutation has an allele frequency close to 100%, pointing to the fixation of resistance-associated variant. The allele frequency of MmpL5 Asn772Thr was 40% and for AtpE Glu61Asp was 60%, resulting in a total of 100%. Therefore, we could speculate the existence of 2 strains, 1 with the simultaneous presence of MmpR5(Ile67fs) and AtpE(Glu61Asp) and the other with MmpR5(Ile67fs) and MmpL5(Asn772Thr). If substitution in MmpL5 decreases susceptibility to bedaquiline, both strains would survive better during bedaquiline treatment and have higher MICs than the parental mutant *mmpR5* strain.

**Figure 1:**
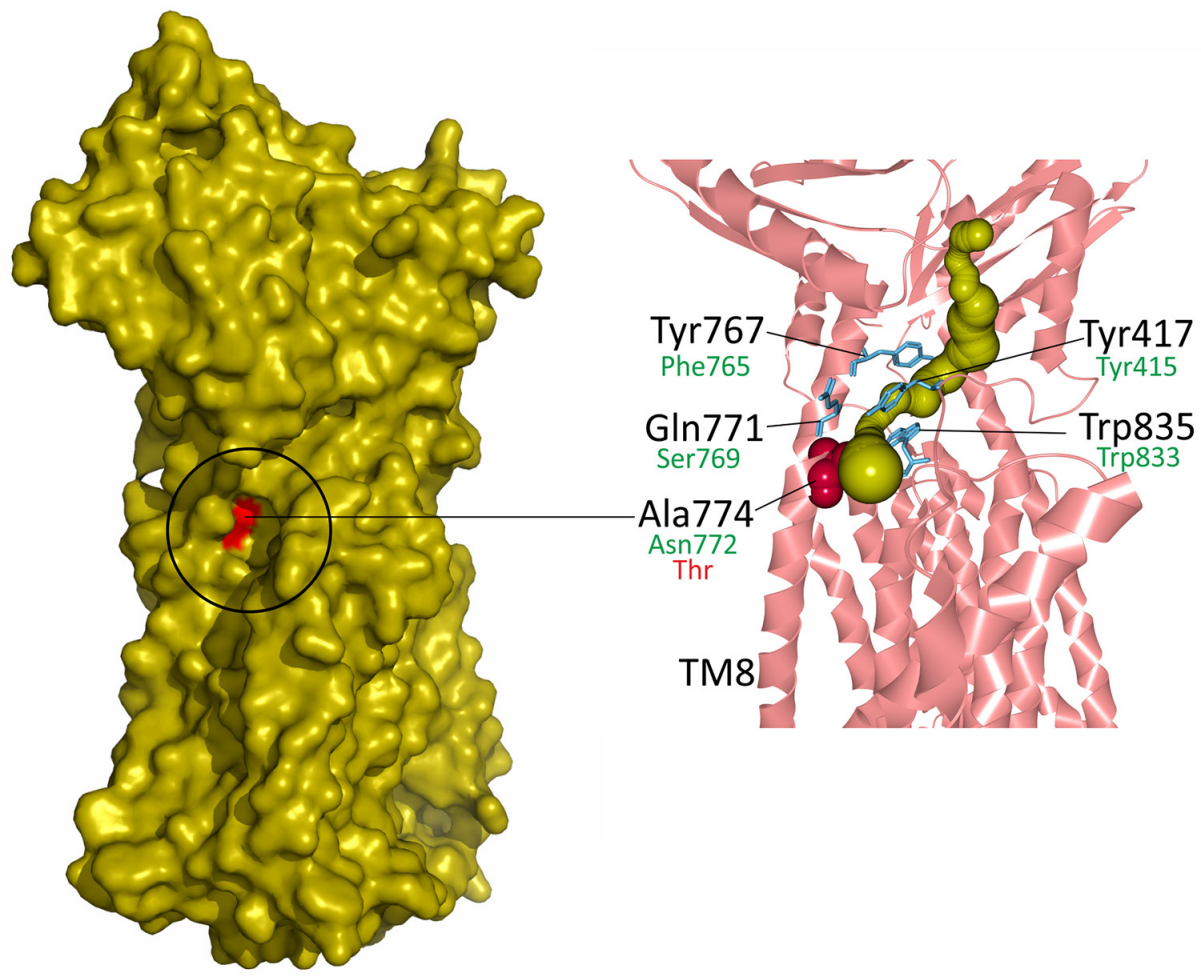
Mapping of the candidate substitution Asn722Thr on the atomic model of *Mycobacterium smegmatis* MmpL5 transporter (PDB: 9B46) in study of high prevalence of *atpE* mutations in bedaquiline-resistant *M. tuberculosis* isolates, Russia. The proposed channel inlet is indicated on the surface model of the MmpL5. The channel model (olive) is shown on the cartoon model of the MmpL5 fragment. Red coloring and text indicates Ala774, located in the transmembrane domain TM8. Green text indicates residues and numbering for *M. tuberculosis*.

Recently, the cryo-EM structure of MmpL5 from *M. smegmatis* was determined, and possible root of transport of mycobactin and other molecules was proposed on the basis of channel prediction, molecular modeling, and docking calculations (*38*). Of note, Ala774 of the *M. smegmatis,* which corresponds to Asn772 of *M. tuberculosis*, is located in the transmembrane domain TM8, responsible for opening and closing of the channel (Figure 1). More precisely, Ala774 is located in the cavity that forms the channel inlet and the narrowest region of the channel together with Tyr767, Gln771, Tyr417, and Trp835, highlighted in the study (*38*). This model should nevertheless be used with caution, taking into account the MmpS5-guided trimerization of MmpL5 and anchoring of the complex in membrane, which is necessary for bedaquiline efflux (*39*).

Mutations in the *mmpL5* are not frequent; however, MmpL5/S5 efflux does not appear to be indispensable, since a substantial number of frameshift mutations were found in different sublineages of *M. tuberculosis* (*19*), and cell wall morphology is not affected in strains with deletion of *mmpL5* or *mmpS5* (*39*). According to the WHO, mutation in the same codon was found earlier; MmpL5(Asn772Ser) has uncertain significance in the resistance to bedaquiline and clofazimine (*18*). Two strains with this mutation in the CRyPTIC database have a low bedaquiline MIC of 0.03 mg/L (*21*).

More notably, the loss-of-function mutation of *mmpL4* was found in a bedaquiline-resistant isolate together with frameshift in the *mmpR5*. Both the MmpL5/MmpS5 and MmpL4/MmpS4 transporter complexes secrete iron-scavenging siderophores mycobactin and carboxymycobactin (*10*). Although the role of MmpL5/MmpS5 in bedaquiline and clofazimine resistance is well established as the export pump of the drugs, mutations in *mmpS4/L4* were not previously associated with bedaquiline resistance. However, a recent study has shown that deletion of *mmpS4* or *mmpL4* increases bedaquiline MIC from 2 to 4 times in vitro, probably because of the compensatory upregulation of the *mmpS5*-*mmpL5* operon (*40*). The 91% allele frequency of the frameshifting variant of *mmpL4* found in isolate #2 points to its recent emergence.

To confirm the significance of our findings, we retrospectively analyzed the genomes of clinical isolates of patients treated with bedaquiline from our previous reports (*9*,*12*). Indeed, in a series of consequent isolates from a patient who was treated with a bedaquiline-containing regimen, a similar loss-of-function mutation of *mmpL4* caused by frameshift in the 341 codon emerged (Table 3). The strain already had a high bedaquiline MIC caused by the presence of amino acid substitutions in AtpE and MmpR5. However, in the 2 latest isolates, the emergence of the mutation in *mmpL4* was accompanied by a further increase in bedaquiline MIC, proportional to the allele frequencies, thus confirming the effect of loss-of-function mutations in the *mmpL4* gene.

**Table 3:** Bedaquiline MIC values and allele frequencies of mutations in genes associated with bedaquiline resistance for sequential isolates for patient from study of high prevalence of *atpE* mutations in bedaquiline-resistant *Mycobacterium tuberculosis* isolates, Russia*

Gene or intergenic region	Amino acid substitution	Isolate Af.102, day 236	Isolate Af.103, day 406	Isolate Af.104, day 580	Isolate Af.105, day 672	Isolate Af.106, day 1,489
Bedaquiline MIC (7H11)		0.25	0.25	0.25	1	1
Bedaquiline MIC (MGIT 960)		2	4	4	8	16
AtpE	Ala63Val	99%	100%		99%	100%
MmpR5 (Rv0678)	Leu142Arg	100%	99%	98%	100%	100%
MmpL4	Val341fs				75%	98%

*Patient described by Peretokina et al. (*12*). Percentages indicate relative number of reads with mutations.

The third gene, which was also supposed to have an effect on bedaquiline resistance, was the *atpB* gene encoding *a* subunit of the ATP synthase. This subunit is a part of the stator, lying in tight contact with the rotor, which consists of 9 identical *c* subunits encoded by the *atpE* gene. Moreover, in the ATP synthase operon *atpB* gene is positioned right upstream of *atpE*, and mutations in bedaquiline resistant isolates were already described (*24*). However, those mutations were found close to the 3′ end of the gene (codons 222, 244, and 250) and were supposed to alter the transcription of the downstream *atpE* gene.

**Figure 2:**
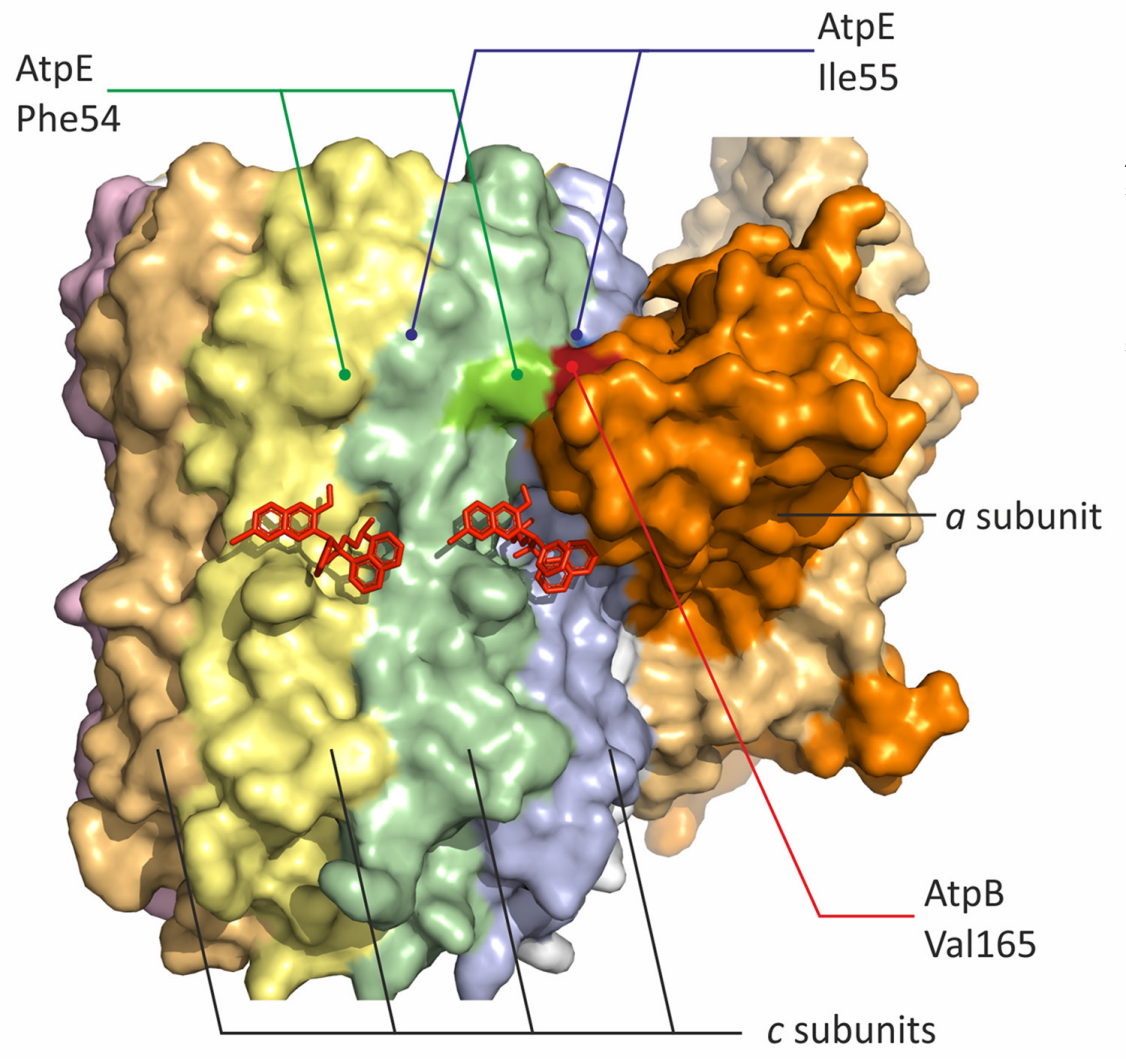
Mapping of the candidate substitution Val165Ile in AtpB on the atomic model of *Mycobacterium tuberculosis* ATP synthase (PDB: 8J0S) in study of high prevalence of *atpE* mutations in bedaquiline-resistant *M. tuberculosis* isolates, Russia. Identical *c* subunits of the rotor (encoded by the *atpE* gene) are shown with distinct colors.

We identified the substitution of Val165, which is located at the *a*/*c* interface close to the lagging binding site of bedaquiline in ATP synthase (*41*). It interacts with Phe54 and Ile55 of the *c* subunit, and substitution for a larger Leucine could change the structure substantially (Figure 2). This strain also had a loss-of-function mutation of *mmpR5* and was susceptible to bedaquiline. Therefore, the particular role of AtpB Val165Ile cannot be established from our data.

The CRyPTIC database contains 18 isolates with the neighboring AtpB(Thr166Met) (*21*). They belong to the same clone inside lineage 3 isolated in different laboratories (Appendix Figure). They do not contain mutations in genes associated or involved in bedaquiline resistance: *atpE*, *mmpR5*, *pepQ*, *mmpL5*, *mmpS5*, *mmpL4*, and *mmpS4*. Furthermore, no other isolates with other mutations in *atpB* were found in the CRyPTIC study. The MIC values of those isolates were slightly shifted toward higher values compared with the entire set of strains, pointing to the possible involvement of this substitution in decreased susceptibility to bedaquiline (Table 4).

**Table 4:** Bedaquiline MIC values for isolates from the CRyPTIC study with mutation in *atpB* gene in study of high prevalence of *atpE* mutations in bedaquiline-resistant *Mycobacterium tuberculosis* isolates, Russia

Isolates	Bedaquiline MIC, mg/L											
	<0.008	<0.015	0.015	0.03	0.06	0.12	0.25	0.5	1	>1	2	>2
All isolates	337	1199	851	3405	3082	603	194	54	26	5	3	5
AtpB (Thr166Met)					3	8	4		3			

The frequencies of mutations, associated with resistance to other drugs, also changed in the series obtained from 1 patient. Therefore, the substitution in the *alr* gene encoding alanine racemase (*42*,*43*), associated with resistance to D-cycloserine, has disappeared in the third isolate #9c, which probably points to its high fitness cost. However, another mutation in the *gabD2* gene, previously associated with resistance to D-cycloserine (*44*), emerged. The lineage 4 strains with the loss-of-function *gabD2* mutation were more frequently identified in patients in the epidemiologic study from Colombia (*45*), thus associated with success in transmission, which could be affected also by reduced susceptibility to drugs.

Mutated variants of 2 genes, *rv2690c* and *ceoC*, previously associated with resistance to pyrazinamide (for *rv2690c*) (*46*) and isoniazid (for *ceoC*) (*47*), emerged in the latest isolate. Those genes could be responsible for virulence and host adaptation also (*48*,*49*). Other genes associated with virulence, such as *rv2298* and 2 PPE genes, also changed the frequencies of mutated alleles in sequential isolates.

Research of the microevolution of the pathogen within the host has allowed for clarity into genetic traits related to resistance and host adaptation, thus complementing the massive body of genome-wide association studies. However, it is hard to discern whether the mutations emerged internally, under selective pressures from treatment or the immune system of the host, or are neutral, from genetic drift caused by spread in the general population. In addition, the exact pressure is not known because of poor adherence to treatment in most chronic cases and individual variability in pK/pD (*50*).

Appendix Additional information about high prevalence of *atpE* mutations in bedaquiline-resistant *Mycobacterium tuberculosis* isolates, Russia
