# Supplementary material for: High Prevalence of atpE Mutations in Bedaquiline-Resistant Mycobacterium tuberculosis Isolates, Russia
Source: Emerg Infect Dis. 2025 Mar;31(3):526–35. doi: 10.3201/eid3103.241488 (PMC11878324; doi:10.3201/eid3103.241488)
Supplement: Supplementary file 1 — Appendix Additional information about high prevalence of atpE mutations in bedaquiline-resistant Mycobacterium tuberculosis isolates, Russia [file 24-1488-Techapp-s1.pdf]

Article DOI: https://doi.org/10.3201/eid3103.241488

EID cannot ensure accessibility for supplementary materials supplied by authors. Readers who have difficulty accessing supplementary content should contact the authors for assistance.

High Prevalence of *atpE* Mutations in Bedaquiline-Resistant *Mycobacterium tuberculosis* Isolates, Russia

Appendix

IDs of isolates with AtpB (Thr166Met):

site.10.subj.YA00106062.lab.YA00106062.iso.1

site.10.subj.YA00132748.lab.YA00132748.iso.1

site.10.subj.IF02733486.lab.IF02733486.iso.1

site.17.subj.K0039.lab.123–20–0039–1000.iso.1

site.17.subj.K0110.lab.123–20–0110–1020.iso.1

site.17.subj.K0119.lab.123–20–0119–1000.iso.1

site.17.subj.K0068.lab.123–20–0068–1000.iso.1

site.10.subj.CG03914604.lab.CG03914604.iso.1

site.20.subj.SCH7774797.lab.YA00131099.iso.1

site.20.subj.SCH7837445.lab.YA00131170.iso.1

site.20.subj.SCH8437501.lab.YA00134607.iso.1

site.20.subj.SA00403702.lab.YA00135281.iso.1

site.20.subj.SCH8167837.lab.YA00134199.iso.1

site.10.subj.XD02142052.lab.XD02142052.iso.1

site.10.subj.YA00042340.lab.YA00042340.iso.1

site.10.subj.YA00127802.lab.YA00127802.iso.1

site.10.subj.BC01599194.lab.BC01599194.iso.1

site.10.subj.DF00772006.lab.DF00772006.iso.1

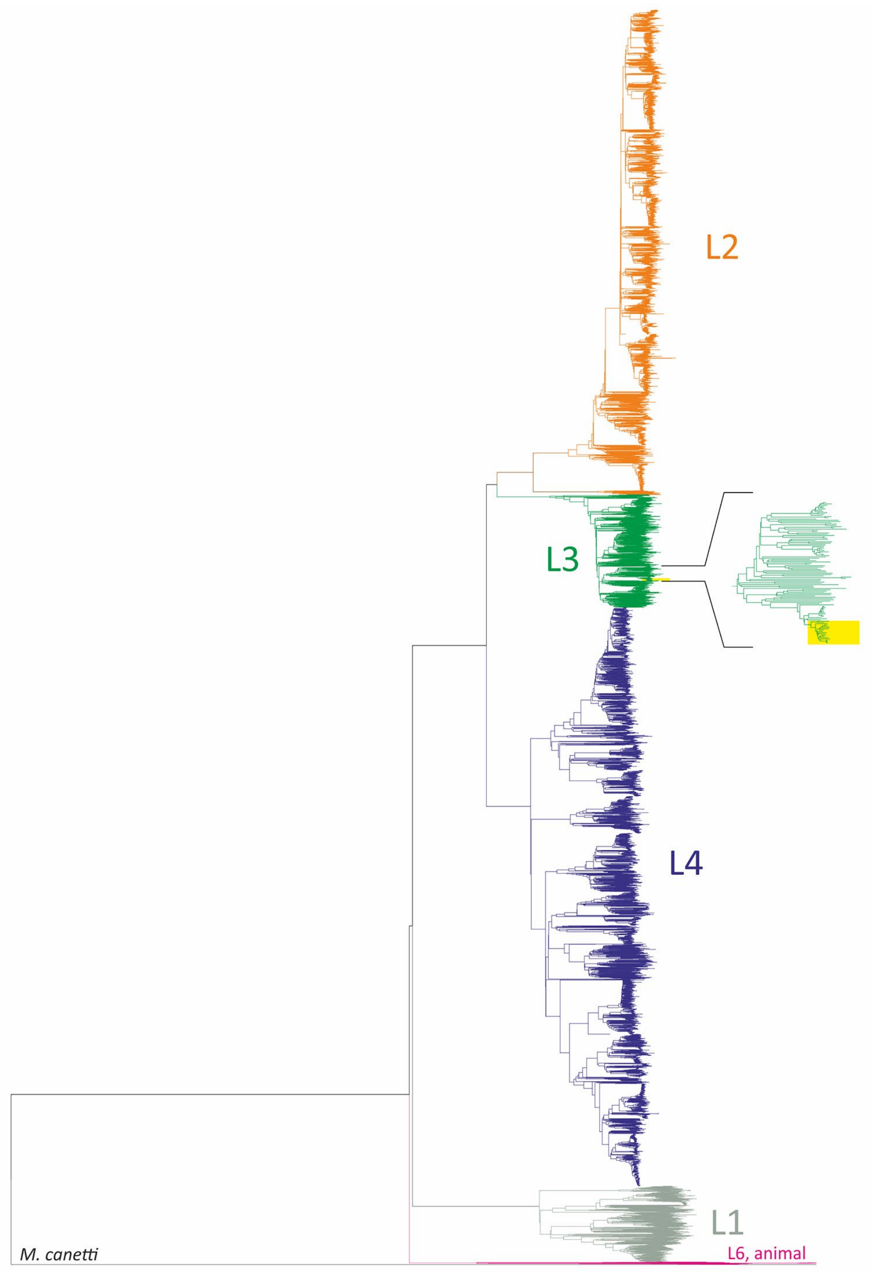

Appendix Figure. Phylogenetic tree of clinical *M. tuberculosis* isolates from the CRyPTIC study rooted on *M. canetti*. Main lineages are color-coded. Yellow bar designates the position of 18 isolates with AtpB (Thr166Met) substitution.
